# Supplementary material for: Contour: A semi-automated segmentation and quantitation tool for cryo-soft-X-ray tomography
Source: Biol Imaging. 2022 May 17;2:e3. doi: 10.1017/S2633903X22000046 (PMC7612748; doi:10.1017/S2633903X22000046)
Supplement: Supplementary file 1 [file S2633903X22000046sup001.docx]

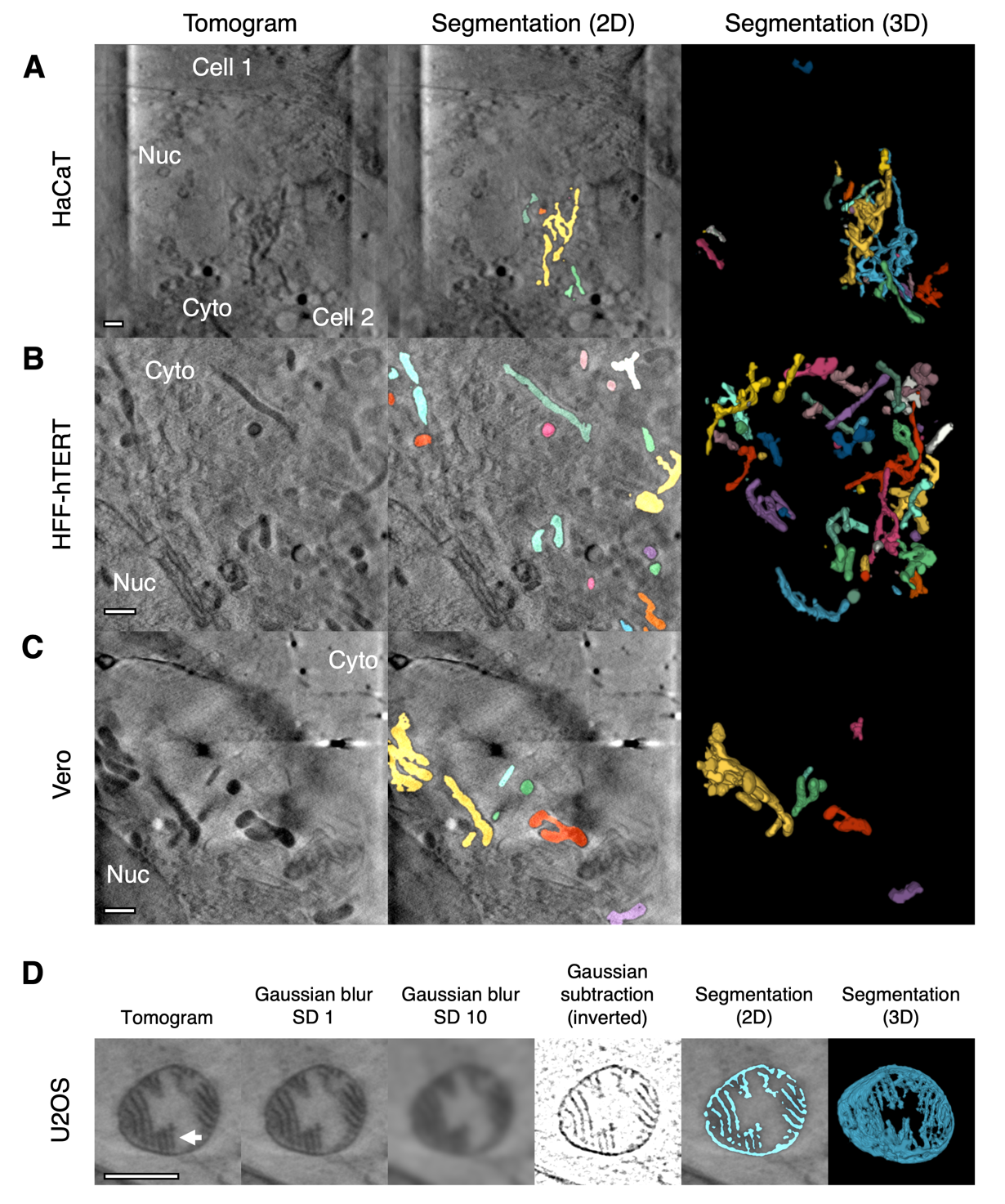


**Supplementary Figure 1. Segmentation of pleomorphic mitochondria from multiple cell types.** (A) Mitochondria were segmented from a cultured human keratinocyte (HaCaT cell) captured using a 40 nm zone plate objective. (B) Mitochondria were segmented from a human foreskin fibroblast immortalised by a human telomerase reverse transcriptase (HFF-hTERT cell) using a 25 nm zone plate objective. (C) Mitochondria were segmented from a green monkey kidney epithelial cell (Vero cell) using a 25 nm zone plate objective. (D) Segmentation of detectable cristae (arrow) in a swollen mitochondrion with a light lumen from a U2OS cell captured using a 25 nm zone plate objective. Although it is possible to segment cristae using *Contour*, a few additional steps are needed to compensate for the narrow widths of the cristae. Using Fiji, a 200×200 voxel section was cropped from a tomogram and scaled up in the XY plane by 2.5× to increase the number of voxels along the width of the cristae so that a width restriction could be effectively used during segmentation. Gaussian blurs with 1 and 10 standard deviations (SD) were applied and the SD 1 Z stack was subtracted from the SD 10 Z stack to digitally increase the signal-to-background ratio^(31)^. The subtracted image was inverted and local segmentations were run in *Contour* using a width restriction of 3 voxels*.* Here, the 2D segmentation is overlayed onto the original section from the tomogram, rather than the subtracted image used to create it. A smoothing factor of 3 and a Gaussian blur with an SD of 1 was applied to the segmented volume in *Contour* and a 3D rendering was produced using 3D Viewer in Fiji^(2)^. Scale bars = 1μm. Nuc, nucleus. Cyto, cytoplasm.

**Supplementary Table 1. Timetable of processes in *Contour.*** Most processes are rapid but local segmentations may take up to 2 hours depending on the number of features that need to be segmented. Under these circumstances, local segmentations will still be faster than manual segmentation tools.

| **Process** | **Estimated Time** | **Factors affecting length of time** |
| --- | --- | --- |
| Global segmentation | 5–10 minutes | - Number of image projections in the Z stack - Number of structures that will be segmented |
| Local segmentation | 0.5–2 hours | - Number of elements that will be segmented - Whether global segmentation was skipped |
| Differentiate elements | 5-10 minutes | - Number of segmented elements |
| Quantitate volumes (automatic) | Seconds |  |
| Quantitate widths (optional) | 0.5–2 hours | - Number of segmented elements - Number of surface voxels for each segmented element |
| Filter elements | 5–10 minutes | - Threshold volume - Number of elements with a volume below the threshold |
| Smoothing | Seconds |  |
| Gaussian blur | Seconds |  |
